# Accelerating Therapeutics for Opportunities in Medicine: A Paradigm Shift in Drug Discovery

**DOI:** 10.3389/fphar.2020.00770

**Published:** 2020-06-30

**Authors:** Izumi V. Hinkson, Benjamin Madej, Eric A. Stahlberg

**Affiliations:** Frederick National Laboratory for Cancer Research, Frederick, MD, United States

**Keywords:** artificial intelligence, machine learning, drug discovery and development, data science, *in silico* modeling

## Abstract

Conventional drug discovery is long and costly, and suffers from high attrition rates, often leaving patients with limited or expensive treatment options. Recognizing the overwhelming need to accelerate this process and increase success, the ATOM consortium was formed by government, industry, and academic partners in October 2017. ATOM applies a team science and open-source approach to foster a paradigm shift in drug discovery. ATOM is developing and validating a precompetitive, preclinical, small molecule drug discovery platform that simultaneously optimizes pharmacokinetics, toxicity, protein-ligand interactions, systems-level models, molecular design, and novel compound generation. To achieve this, the ATOM Modeling Pipeline (AMPL) has been developed to enable advanced and emerging machine learning (ML) approaches to build models from diverse historical drug discovery data. This modular pipeline has been designed to couple with a generative algorithm that optimizes multiple parameters necessary for drug discovery. ATOM's approach is to consider the full pharmacology and therapeutic window of the drug concurrently, through computationally-driven design, thereby reducing the number of molecules that are selected for experimental validation. Here, we discuss the role of collaborative efforts such as consortia and public-private partnerships in accelerating cross disciplinary innovation and the development of open-source tools for drug discovery.

## Introduction

Preclinical drug discovery typically takes five and a half years and accounts for about one third of the cost of drug development (Paul et al., 2010). The process is largely empirical with a sequential, iterative approach to optimizing key drug discovery parameters—efficacy, pharmacokinetics (PK), safety, and developability. Millions of molecules are tested, thousands are produced, and most fail to progress in preclinical or clinical settings (Shannon Decker and Atkinson, 2007; Mohs and Greig, 2017). Furthermore, translation from R&D to the clinic is insufficient with a success rate of less than 10%, and safety liabilities and poor efficacy cited as the main causes of attrition (Miller et al., 2017; Lowe, 2019).

Patients are waiting for the field of drug discovery to innovate new processes that will help improve the success rate of pharmaceutical development, lower drug costs, and get medicines to the clinic more quickly. With the average cost of developing a new molecular entity at over $2 billion, in large part due to the costs of failures, researchers are challenged to work outside the conventional slow, sequential, and costly drug development paradigm to better meet the urgent needs of patients (Kramer et al., 2007; Munos, 2009; Mullin, 2014; DiMasi et al., 2016). To increase the generation of successful new molecular entities, a number of groups have called for more innovation around the culture of and approach to drug discovery (Munos, 2006; Papadaki and Hirsch, 2013; Parekh et al., 2015). In particular, because so much of the cost of development stems from the cost of failures, approaches that improve our ability to distinguish early which molecules will ultimately succeed can have a disproportionate impact on improving the output of new medicines illustrate the potential for accelerating drug discovery through artificial intelligence (AI)-driven approaches (Ringel et al., 2013).

The demonstrations of ML for polypharmacological drug design, deep neural nets for predicting quantitative structure-activity relationships (QSAR), and generative molecular design through the use of variational autoencoders and generative adversarial networks (Besnard et al., 2012; Ma et al., 2015; Blaschke et al., 2018) hold great promise. To this end, significant interest has been raised in the application of approaches that combine AI, simulation, and experimentation to drug discovery (Vamathevan et al., 2019). Recognizing the compelling need for a paradigm shift in drug development, the ATOM consortium was established in October 2017^1^. ATOM's founders, the Frederick National Laboratory for Cancer Research (FNLCR, on behalf of the National Cancer Institute), Lawrence Livermore National Laboratory (LLNL, on behalf of the Department of Energy), GSK (GlaxoSmithKline), and the University of California, San Francisco (UCSF), have joined forces to leverage resources toward the common goal of benefiting patients. ATOM is applying an integrated approach to combine capabilities such as high-performance computing, human-relevant *in vitro* experimentation, data-driven and mechanistic modeling, and curation of pharmacological data toward the development of a novel preclinical drug discovery and development platform.

### Drug Discovery Consortia

As the complexity of biomedical research questions has increased, so too has the need to bring together expertise and resources from multiple disciplines and organizations (Cooke et al., 2015). Consequently, several articles by thought leaders have called for more collaboration in the drug development process (Altshuler et al., 2010; Dahlin et al., 2015; Alteri and Guizzaro, 2018; Takebe et al., 2018; Chaturvedula et al., 2019). Open innovation and open-source research strategies which emphasize the value of collaboration and use of both internal and external information, are creating the opportunity for the drug research and development industry to leverage know-how from across organizations (Munos, 2006; Hunter and Stephens, 2010; Owens, 2016). Cross-industry collaboration is particularly important in the application of computational approaches to drug discovery, where for instance, most companies have one or fewer drugs approved per year, far too small a sample size to support these approaches (Munos, 2009). The advantages of bringing together organizations into public-private partnerships (PPP) and consortia include not just scale, but also new-found agility and increased creativity alongside risk reduction and cost sharing (Papadaki and Hirsch, 2013; Slusher et al., 2013; Rosenberg, 2017; Kuchler, 2019). In fact, the US Food and Drug Administration (FDA), acknowledges the critical role of PPPs and consortia with respect to the innovation and modernization of medical product development (Maxfield et al., 2017).

One notable example of cross-sector collaboration is the Merk Molecular Activity Challenge^2^ where the pharmaceutical company provided contestants with a training set of molecular descriptors and activities and a test set of descriptors only, and spurred the development of innovative ML methods for QSAR (Ma et al., 2015). In the last 2 years, new academic-industry consortia projects have emerged, focusing on applications of ML in drug discovery. The Machine Learning for Pharmaceutical Discovery and Synthesis Consortium, with membership from three Massachusetts Institute of Technology departments and several leading pharmaceutical companies, focuses on the application of ML to automate drug discovery and synthesis^3^. Summer 2019 saw the start of a new Innovative Medicines Initiative collaborative project led by Janssen, dubbed Machine Learning Ledger Orchestration for Drug Discovery (MELLODDY)^4^ (Kuchler, 2019). With a 3-year timeframe, the MELLODDY project focuses on employing federated ML to foster sharing data insights while preserving organizational intellectual property. Pharmaceutical industry participants will train models on their own proprietary data and share those models to increase the impact of AI and ML in the industry.

As an open consortium backed by major public entities, the Department of Energy, the National Cancer Institute, and the University of California Office of the President, as well as pharmaceutical leader GSK, the Accelerating Therapeutics for Opportunities in Medicine consortium (ATOM) is committed to creating new tools for drug discovery that can be shared broadly and benefit the public good. Computational approaches to drug design hold the potential to drastically improve the field's ability to generate novel drugs for patients in need. Harnessing advances in computational power and AI, ATOM is building a new, comprehensive, integrated platform for efficient molecular property prediction, optimization, and design. Drawing from team science, open innovation, and open-source concepts, the ATOM platform combines ML, simulation, and experimentation to generate novel drug candidates more rapidly than traditional approaches. ATOM's current scope focuses within the area of preclinical drug discovery, but its outcomes aim to benefit not only the member organizations and their immediate stakeholders, but the biomedical community at large including academicians, start-ups, private industry, clinicians, and patients.

### AI-Driven Drug Discovery

Drug discovery is relying increasingly on computational and AI-driven methods. Collaborative efforts that combine scientific know-how and computational power are being stood up to incubate innovative methods while sharing risk and accelerating progress. In the past decade significant advances have been made to accelerate the drug discovery process such as the development of computational and AI-based methods for virtual screening and *in silico* drug design. Moving beyond structure-based approaches and virtual screens, several seminal publications have demonstrated the use of generative adversarial networks and variational autoencoders for *de novo* drug design (Kadurin et al., 2017; Olivecrona et al., 2017; Gómez-Bombarelli et al., 2018; Merk et al., 2018; Polykovskiy et al., 2018; Putin et al., 2018; Segler et al., 2018; Ståhl et al., 2019; Hong et al., 2020). For example, a recently published deep generative model demonstrated the design of small-molecule drug candidates for discoidin domain receptor 1 prioritizing synthetic feasibility, efficacy, and uniqueness with respect to known small molecules, showcasing the ability to rapidly discover drugs at low cost (Zhavoronkov et al., 2019).

#### Collaborative AI-Driven Drug Discovery at ATOM

The promise of AI-driven drug design carries with it, several challenges—the need for appropriate datasets, ability to generate and test evolving biological hypotheses, multi-parameter optimization, reduction in design-make-test-analyze cycle times, and adaptability of research culture (Schneider et al., 2020). ATOM is tackling these challenges through the collaborative development of a preclinical, open-source, small-molecule drug discovery platform (Chaturvedula et al., 2019). The initial stages have focused on building computational infrastructure, curating preclinical data from both GSK and public sources, and creating and testing data-driven modeling capabilities.

ATOM has developed a data-driven modeling pipeline capable of rapidly building and optimizing ML models for bioassay activity and molecular property predictions. This modeling pipeline is important for developing predictive models for public and private pharmaceutical assay datasets. While ML-based techniques to predict drug properties from structures are regularly used in the field of computational drug design, there remains a need for an automated modular pipeline for common modeling tasks. Some key features for such a software package are to enable reproducibility, incorporate new models, support a variety of chemical representations, allow for hyperparameter optimization, and validate predictive performance (Dahl et al., 2014; Gilmer et al., 2017; Feinberg et al., 2018; Yang et al., 2019).

Existing commercial pipeline tools such as BIOVIA Pipeline Pilot are limited in their customizability and can be cost prohibitive to small academic research groups and start-up companies^5^. On the other end of the spectrum, open-source pipeline tools such as KNIME are useful as GUI-based platforms for data processing, model fitting, and analysis, (Berthold, 2008) but have yet to demonstrate the suitability for large scale model generation.

#### The ATOM Modeling Pipeline (AMPL)

AMPL^6^, or the ATOM Modeling Pipeline, extends the popular DeepChem^7^ library and supports ML and molecular featurization tools (Minnich et al., 2020). AMPL is implemented as a Python library that integrates with existing data science ecosystems and utilities. AMPL automates and optimizes many common ML model fitting tasks that are performed for pharmaceutical datasets including model fitting, validation, and prediction. AMPL allows researchers to reproducibly train and test models, incorporate new models, and provide utilities for automated dataset characterization, model validation, and uncertainty quantification. AMPL is designed to be a versatile library that can interface with many services and tools.

AMPL allows users to build *in silico* models based on molecular properties to aid in drug discovery. With an initial focus on safety and pharmacokinetic modeling, AMPL has been extensively tested on activity and property assay datasets. In preparation for the initial release of the pipeline, 11,552 regression and classification models were built to evaluate data splitting algorithms, model types, and feature types (Minnich et al., 2020). AMPL supports a wide variety of dataset splitting algorithms for validation and testing, including random splits, Butina clustering, scaffold splits, and temporal splits. AMPL uses models from scikit-learn and DeepChem including random forest, XGBoost, fully connected neural network, and graph convolution neural network models. Small molecules were represented as SMILES strings using the RDKit cheminformatics library and the molecule validation and standardization tool, MolVS. AMPL's data curation module was applied to datasets to filter out compound assay values with wide variability, and to characterize the datasets with Tanimoto distances between chemical fingerprints or Euclidean distances between descriptor feature vectors. Several featurization approaches were compared including Extended Connectivity Fingerprints (ECFP), DeepChem graph convolution latent vectors, Mordred chemical descriptors, and Molecular Operating Environment (MOE) descriptors. Due to the modular nature of AMPL's implementation, extensions to the pipeline are available for additional splitting algorithms, model types, and feature types.

Hyperparameter optimization is an important task for cheminformatics ML model fitting that may improve model predictive performance. AMPL supports basic hyperparameter optimization functions including searches using basic linear grids, logistic grids, random searches, and user-specified searches. Model fitting for safety and pharmacokinetic parameters used AMPL's hyperparameter optimization module to explore model parameter combinations. Generally, hyperparameter optimization improved predictive performance on properties of external test sets except for certain cases with limited data or ECFP featurization.

AMPL automatically calculates standard model performance metrics for regression and classification models. The regression performance statistics include R^2^, mean absolute error, and mean square error to evaluate the level of agreement between the model predicted values and actual experimental ground truth values. AMPL also includes classification performance metrics such as precision and recall, area under the precision-recall curve (PRC-AUC), negative predictive value, cross entropy, and accuracy metrics. As previously described, model prediction uncertainty was calculated for several of PK datasets for comparison with model prediction error (Minnich et al., 2020). AMPL enables this type of uncertainty quantification analysis toward better understanding model predictions, uncertainty, and error.

AMPL is open-source, modular, and flexible, allowing for additions or extensions as needed. This makes data-driven modeling using modern ML libraries accessible to the wider scientific community including academic or government laboratories and small companies. AMPL is now available for download on Github^8^. The website includes detailed library documentation as well as example Jupyter notebooks to learn to use the pipeline.

#### AMPL Validation

Bioassay data, specifically the half-maximal effective drug concentration (EC50), and the half-maximal inhibitory drug concentration (IC50), of known hepatic, central nervous system, cardiovascular, and cellular toxicity safety liabilities were used to benchmark safety models. Models were fit for assays such as BSEP, β2 adrenoceptor, muscarinic acetylcholine receptor, dopamine D2, voltage-gated potassium channels, and phospholipidosis induction. For each assay type, model hyperparameters were optimized resulting in 2,130 classification models with thresholds appropriate set for each assay. As described by Minnich et al, the predictive performance of the classification models was evaluated using common validation statistics including receiver operating characteristic area under the curve (ROC AUCs) built on safety datasets. Predictive performance varied based on assay type, dataset size, dataset split type, feature type, and model type, but overall produced many useful models for pharmaceutical safety properties (Minnich et al., 2020).

A diverse set of pharmacokinetic data including blood-to-plasma ratio, plasma protein binding, *in vivo* clearance, volume of distribution, hepatocyte clearance, and microsomal clearance, logD was used to fit predictive models with AMPL (Minnich et al., 2020). Nine thousand four hundred twenty-two regression models were fit for all the assay types and corresponding model parameters were evaluated for improvements to predictive performance as described by Minnich et al. General trends between different training and test splits, feature types, and model types were examined. When using neural network models with calculated descriptors for many of these PK datasets, model predictions with MOE descriptors were slightly better than predictions with open-source Mordred descriptors. Several PK datasets with larger numbers of measurements (10,000 or more) benefitted from DeepChem's graph convolutional neural network models with better predictions compared to experiment than ECFP or calculated descriptors. For smaller PK datasets, random forest models with MOE descriptors had slightly better performance than other feature and model combinations (Minnich et al., 2020).

AMPL is designed to automatically and rapidly build and evaluate cheminformatics models. Automation of deep learning model training, parallelized hyperparameter search, performance benchmarking, and data and model storage are essential for reproducible ML predictions in drug discovery. Given the wide range of activity and property assay types, the validation performed by Minnich et al. demonstrate there is no single best model fitting approach for every dataset. This underscores the need to rapidly search and fit predictive models for new datasets enabled by the AMPL software suite.

Two examples of model fitting on publicly accessible datasets are available with the AMPL repository. Each example describes a general method of curating datasets, fitting a ML model, and using the created model for new predictions. Example code is included to download the datasets from their original source, perform basic curation on the datasets, train a model on the curated datasets, and then load the fitted model for prediction on a withheld test set. In the first example, AMPL mimicked a DeepChem example model by fitting a model to a public aqueous solubility dataset using DeepChem's graph convolutional neural network model (Delaney, 2004). In a second example, AMPL was used to fit a predictive neural network model using Mordred descriptors for human liver microsomal clearance from a public PK dataset (Wenzel et al., 2019). The entire process of data curation to analysis and visualization for these sample datasets is automated and reproducible with the AMPL library and tools.

AMPL models can be applied toward related compounds to rapidly predict bioassay activity or safety and pharmacokinetic properties. In the context of ATOM, AMPL is a key component in the overall mission to accelerate the drug discovery process.

## Conclusions

Given heavy reliance on expensive and lengthy experimentation, the field of drug discovery is increasingly integrating both computational and AI-driven methods for virtual screening and *in silico* drug design. Further, the application of deep neural network architectures in generative design in conjunction with data-driven and mechanistic modeling for functional property prediction and an *in silico* framework for rapid lead optimization will drastically change how drug discovery is done.

Collaborative efforts have been employed in recent efforts to develop new capabilities where risks and required investment have been high. ATOM provides an avenue for collaborative AI-driven drug discovery that results in an open-source framework that broadens availability and an opportunity to raise the level of collaborative drug discovery efforts.

**Figure 1:**
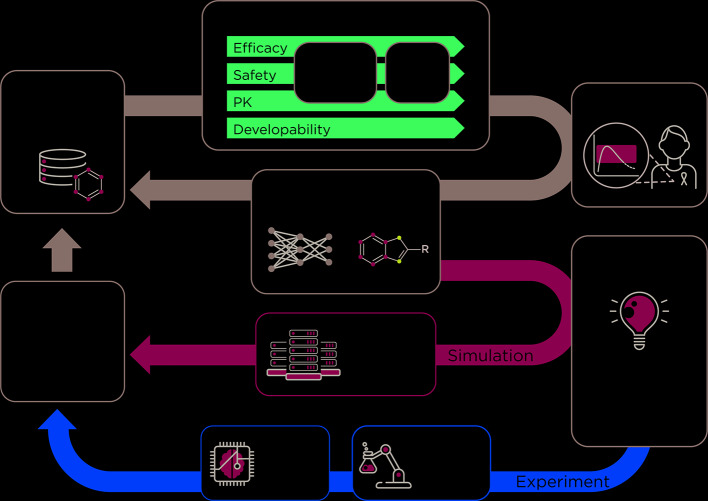
The ATOM preclinical drug discovery workflow. ATOM is developing an active learning drug discovery framework that uses a compound library as input to a property prediction pipeline. The pipeline begins with historic data collected on a working compound library to train machine learning-based models for property prediction. Next, multi-level and systems-level models of efficacy, safety, and pharmacokinetics as well as developability are integrated to generate a set of drug design criteria. These parameters are simultaneously optimized for the generation of novel molecules by the generative molecular design framework. The multi-parameter optimization loop, in grey, can be run for numerous cycles. An active learning approach is used to decide whether a molecular simulation or experiment is needed to improve or validate the models. Data that result from these simulations and experiments are then used to re-train the property prediction models. The result of this workflow is a set of optimized drug candidates.

The AMPL serves as the initial step toward the development of an open-source preclinical drug design platform that will accelerate the process of getting more effective therapies to patients. Future efforts involve extending the modeling capability of AMPL toward the development of an open-source pre-clinical drug discovery platform (**Figure 1**).

### Future Efforts

At ATOM, efforts are underway to integrate current and emerging computational capabilities with active learning in an AI-driven platform. ATOM is creating a generative molecular design framework that integrates predictive models from AMPL and initiates cycles of generative molecular design and multiparameter optimization. The goal of ATOM's generative molecular design framework is to propose novel small-molecule drug candidates with optimized properties based on design criteria such as potency, selectivity, cardiotoxicity, hepatoxicity, solubility, clearance, and synthetic accessibility^9^. New experimental and molecular simulation data will be selectively acquired to support the ML-based approach and will be integrated into the computational pipeline to kick start additional cycles of the molecular design and optimization. The integration of active learning will streamline time-consuming and costly experimentation and will guide the design of novel drug candidates (**Figure 1**). Collectively, these efforts usher in a paradigm shift in drug discovery that emphasizes collaboration, innovation, and the development of open-source tools.

## Author Contributions

IH: manuscript writing and figure design. BM: manuscript writing. ES and ATOM consortium: manuscript and figure revision, approval of final manuscript.

## Funding

This project has been funded in whole or in part with funds from University of California, San Francisco, funds from GlaxoSmithKline, federal funds from the National Cancer Institute, National Institutes of Health, under contract HHSN261200800001E, and federal funds from U.S. Department of Energy, National Nuclear Security Administration under contract DE-AC52-07NA27344. The federal funds include the Cancer Moonshot funds that Congress passed the 21st Century Cures Act in December 2016, authorizing $1.8 billion in funding for the Cancer Moonshot over 7 years. The content of this publication does not necessarily reflect the views or policies of the Department of Health and Human Services nor the Department of Energy; nor does mention of trade names, commercial products, or organizations imply endorsement by the U.S. Government. Frederick National Laboratory is a Federally Funded Research and Development Center operated by Leidos Biomedical Research, Inc., for the National Cancer Institute. Lawrence Livermore National Laboratory is operated by Lawrence Livermore National Security, LLC, for the Department of Energy, National Nuclear Security Administration.

## Conflict of Interest

The authors declare that this study received funding from GSK. The funder had the following involvement with the study: GSK is a founding member of ATOM and has contributed to R&D and consortium activities at ATOM.

## Footnotes

1
atomscience.org

2
www.kaggle.com/c/MerckActivity/data

3
news.mit.edu/2018/applying-machine-learning-to-challenges-in-pharmaceutical-industry-0517

4
www.imi.europa.eu/projects-results/project-factsheets/melloddy

5
www.3dsbiovia.com/products/ collaborative-science/ biovia-pipeline-pilot/

6 github.com/ATOMconsortium/AMPL

7 github.com/deepchem/deepchem

8
github.com/ATOMconsortium/AMPL

9 atomscience.org/abstracts-and-presentations/2019/9/25/generative-lead-optimization-of-de-novo-molecules-case-study-in-discovery-of-potent-selective-aurora-kinase-inhibitors-with-favorable-secondary-pharmacology
